# Amphiphilic Cyclodextrin Nanoparticles as Delivery System for Idebenone: A Preformulation Study

**DOI:** 10.3390/molecules28073023

**Published:** 2023-03-28

**Authors:** Federica De Gaetano, Angela Scala, Consuelo Celesti, Kim Lambertsen Larsen, Fabio Genovese, Corrado Bongiorno, Loredana Leggio, Nunzio Iraci, Nunzio Iraci, Antonino Mazzaglia, Cinzia Anna Ventura

**Affiliations:** 1 Department of Chemical, Biological, Pharmaceutical and Environmental Sciences, University of Messina, Viale Ferdinando Stagno d’Alcontres 31, 98166 Messina, Italy; 2 Department of Engineering, University of Messina, Contrada Di Dio, 98166 Messina, Italy; 3 Department of Clinical and Experimental Medicine, University of Messina, Via Consolare Valeria, 98125 Messina, Italy; 4 Department of Chemistry and Bioscience, Aalborg University, Frederik Bajers Vej 7H, 9220 Aalborg, Denmark; 5 Technical, Economic and Technological Institute “Girolamo Caruso”, Via John Fitzgerald Kennedy 2, 91011 Alcamo, Italy; 6 National Council of Research, Institute of Microelectronics and Microsystems (CNR-IMM), Strada VIII n. 5-Zona Industriale, 95121 Catania, Italy; 7 Department of Biomedical and Biotechnological Sciences (BIOMETEC), University of Catania, Torre Biologica, Via Santa Sofia 97, 95125 Catania, Italy; 8 National Council of Research, Institute for the Study of Nanostructured Materials (CNR-ISMN), URT of Messina c/o Department of Chemical, Biological, Pharmaceutical and Environmental Sciences of the University of Messina, V.le F. Stagno d’Alcontres 31, 98166 Messina, Italy

**Keywords:** amphiphilic cyclodextrins, nanoparticles, idebenone, NMR studies, molecular modeling, in vitro antioxidant activity

## Abstract

Idebenone (IDE), a synthetic short-chain analogue of coenzyme Q10, is a potent antioxidant able to prevent lipid peroxidation and stimulate nerve growth factor. Due to these properties, IDE could potentially be active towards cerebral disorders, but its poor water solubility limits its clinical application. Octanoyl-β-cyclodextrin is an amphiphilic cyclodextrin (ACyD8) bearing, on average, ten octanoyl substituents able to self-assemble in aqueous solutions, forming various typologies of supramolecular nanoassemblies. Here, we developed nanoparticles based on ACyD8 (ACyD8-NPs) for the potential intranasal administration of IDE to treat neurological disorders, such as Alzheimer’s Disease. Nanoparticles were prepared using the nanoprecipitation method and were characterized for their size, zeta potential and morphology. STEM images showed spherical particles, with smooth surfaces and sizes of about 100 nm, suitable for the proposed therapeutical aim. The ACyD8-NPs effectively loaded IDE, showing a high encapsulation efficiency and drug loading percentage. To evaluate the host/guest interaction, UV-vis titration, mono- and two-dimensional NMR analyses, and molecular modeling studies were performed. IDE showed a high affinity for the ACyD8 cavity, forming a 1:1 inclusion complex with a high association constant. A biphasic and sustained release of IDE was observed from the ACyD8-NPs, and, after a burst effect of about 40%, the release was prolonged over 10 days. In vitro studies confirmed the lack of toxicity of the IDE/ACyD8-NPs on neuronal SH-SY5Y cells, and they demonstrated their antioxidant effect upon H_2_O_2_ exposure, as a general source of ROS.

## 1. Introduction

Idebenone (IDE) is an orphan drug currently employed for the treatment of visual impairment in subjects affected by Leber’s hereditary optic neuropathy (LHON) [1], a rare genetic disease responsible for progressive vision loss and caused by a mitochondrial alteration. IDE is also on the Italian market as Mnesis^®^ (Takeda Italia SpA, Rome, Italy) to treat cognitive impairments due to vascular or degenerative neurological diseases [2].

Due to its antioxidant activity [3] and its abilities to prevent lipid peroxidation [4] and activate the electron transfer system of mitochondria [5,6], IDE could potentially be employed to treat various diseases related to defects in the mitochondria respiratory chain. Among them, the treatment of neurological disorders, such as Alzheimer’s Disease (AD), could be of great interest [7]. In fact, in 1986, IDE was marketed in Japan to treat AD, but it was later withdrawn from the market due to a lack of proven effectiveness [8]. Different studies were carried out to prove the efficacy of IDE in the treatment of Friedreich Ataxia [9] and Duchenne muscular dystrophy [10], but contradictory results about its real efficacy [11,12] caused its withdrawal from the market or did not authorize its trade.

The lack of IDE effectiveness is probably due to an insufficient accumulation of the drug into the relevant tissues, such as the brain. Due to its very low water solubility (0.8 mg/100 mL, 25 °C), IDE shows a low oral bioavailability. It is extensively metabolized in the liver and intestinal mucosa, resulting in a very low plasma concentration (about 1%) [13,14], which reduces the therapeutical potential of the drug. To solve the solubility drawback of IDE, cyclodextrins (CyDs) were efficaciously employed [15,16,17]. However, the complexation of the drug into CyDs cannot resolve the fast metabolism of the drug observed after oral administration or the binding of IDE to plasma proteins. Other strategies are needed, and among them, intranasal administration can represent a valid alternative to other administration routes to improve brain biodistribution [18]. The intranasal administration of drugs permits the targeting of the brain by means of olfactory and trigeminal pathways, avoiding first-pass metabolism and improving drug effectiveness [19,20,21]. However, a particulate delivery system with mucoadhesive properties is needed to avoid the fast mucociliary clearance that characterizes the application on nose mucosa [22].

Amphiphilic cyclodextrins (ACyDs) recently received great attention as new drug delivery systems due to their peculiar structure, which permits their self-assembly in water, forming stable supramolecular systems in the form of micelles, nanospheres, nanocapsules or vesicles, without the presence of a surfactant [23,24,25,26,27,28,29,30,31]. They effectively entrap both hydrophilic and hydrophobic drugs, solving the problems of low solubility, stability and bioavailability. Furthermore, modifying the surface of ACyD nanoaggregates via conjugation with recognition molecules allowed for the targeting of anticancer drugs to be achieved, as well as overcoming hemolytic activity on blood cells [32,33,34,35,36,37]. Bilensoy et al. demonstrated the effectiveness of polycationic ACyD nanoaggregates to deliver paclitaxel to breast tumors. These nanoaggregates show a high ability to interact with biomembranes due to their net-positive charge, enabling them to interact with the negatively charged molecules present on membrane cells, i.e., cholesterol, sialic acid or phospholipids [38]. Moreover, polycationic ACyDs demonstrated high potentiality as gene-delivery systems, forming complexes with plasmid DNA and siRNA [39,40,41]. Their biocompatibility and the ability to interact with mucosae made them interesting carriers of delivery drugs through different administration routes.

On these bases, here, we developed an innovative delivery system for the potential intranasal administration of IDE, based on amphiphilic octanoyl-β-CyD nanoparticles (ACyD8-NPs). Interaction studies between IDE and ACyD8 were carried out using UV-vis and NMR spectroscopy. Molecular modeling studies were performed to assess the IDE/ACyD8 arrangement and the energetics of the inclusion complex in water. Molecular dynamics simulations revealed a major role of van der Waals energy contributions in the stability of the host/guest interaction and, in agreement with previously reported studies [42,43], the tendency of ACyD8 to become involved in self-inclusion phenomena. ACyD8-NPs loading IDE (IDE/ACyD8-NPs) were prepared using the nanoprecipitation method and were characterized for their size, morphology and encapsulation parameters. Moreover, in vitro release studies of IDE from IDE/ACyD8-NPs were performed. Finally, the IDE/ACyD8-NPs’ safety and efficacy were evaluated on a neuronal cell model of oxidative stress.

## 2. Results

### 2.1. ACyD8/IDE Interaction Studies

**Figure 1:**
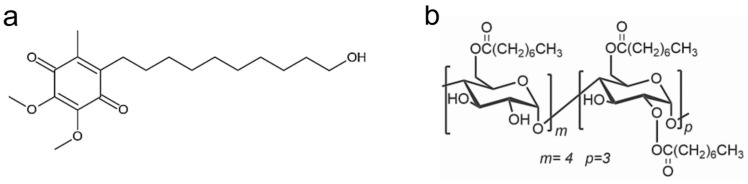
Chemical structure of IDE (**a**) and ACyD8 (**b**).

The interaction between IDE and ACyD8 (Figure 1) was studied as follows: (a) in a solution, using UV-Vis titration to determine the apparent binding constant (Kc) as a measure of the affinity of the drug for the macrocycle, and using NMR analyses to study the supramolecular interaction and to better understand the complexation phenomena and the binding sites; (b) in silico, using molecular dynamics (MD) simulations to elucidate the complex conformations and the best accommodation of IDE in ACyD8.

#### 2.1.1. UV-Vis Titration Studies

**Figure 2:**
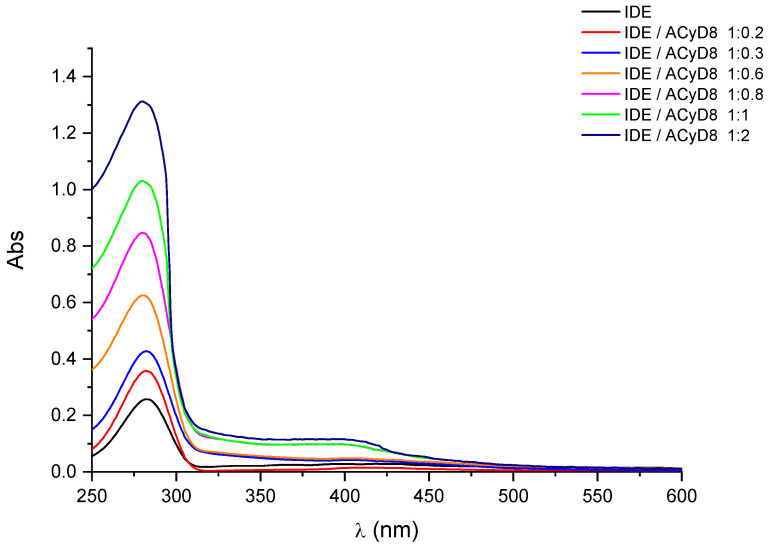
UV-vis spectra of IDE in the presence of increasing concentration of ACyD8. See Experimental Section for details.

In Figure 2, we report the UV-vis spectra of IDE (1 mM) obtained in the presence of an increasing concentration of ACyD8 (from 0 to 2 mM). IDE showed two bands: one at 280 nm, due to the π → π* of the conjugate quinone system, and one with a very low intensity at 408 nm, due to the radical transition of the carbonyl chromophore [44]. A significant hyperchromic effect involved the band at 280 nm, as its intensity progressively increased, enhancing the ACyD8 concentration. A small shift towards lower wavelengths of about 3 nm was also evident. These effects are due to the variation in the local polarity caused by the accommodation of the IDE molecules within the apolar cavity of the macrocycle, and they are due to the formation of new hydrophobic–hydrophobic interactions [45]. Due to the large extension of the ACyD8 cavity, produced by the presence of octanoyl chains on both rims of the macrocycle, the total inclusion of the drug within the cavity is conceivable. Furthermore, a non-inclusion complexation that could involve the exterior surface of the macrocycle, including octanoyl chains, cannot be excluded [46].

**Figure 3:**
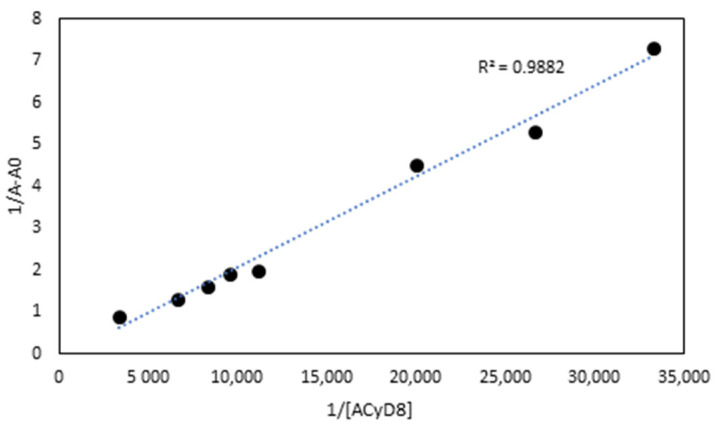
The Benesi–Hildebrand plot of 1/A-A_0_ vs. 1/[ACyD8] obtained from UV-vis data.

UV-vis data were used to determine the apparent binding constant (Kc) of the 1:1 IDE/ACyD8 inclusion complex on the basis of the Benesi–Hildebrand equation [45]. A linear relationship (R^2^ = 0.9882) was obtained for the plot of 1/A-A_0_ versus 1/[ACyD8] (Figure 3), but not for the plot of 1/A-A_0_ versus 1/[ACyD8]^2^ (R^2^ = 0.9676) (plot not shown) demonstrating the presence in solution of a 1:1 inclusion complex. The Kc value determined by the ratio of the intercept on the slope was 19.698 × 10^3^ M^−1^, pointing out a high affinity of IDE for the amphiphilic macrocycle.

#### 2.1.2. NMR Studies

**Figure 4:**
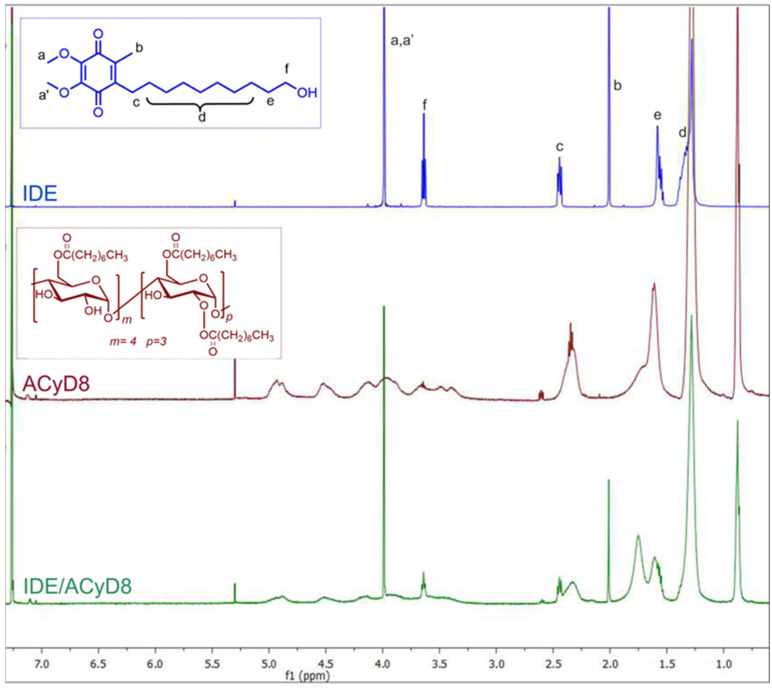
Stacked ^1^H NMR spectra of free IDE (blue trace), ACyD8 (red trace) and IDE/ACyD8 (green trace). In the insets: chemical structures of IDE and ACyD8. Lowercase letters **a**–**f** were used for IDE proton assignment.

**Figure 5:**
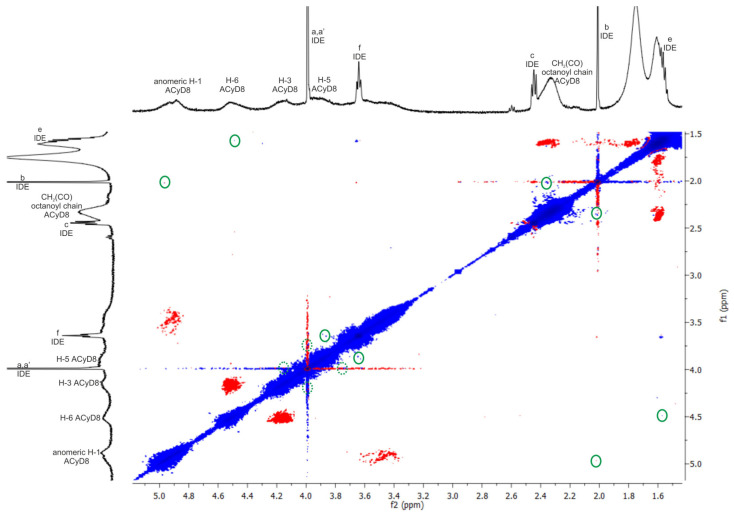
The 2D ROESY spectrum of IDE/ACyD8 complex (CDCl_3_, at 25 °C). Inter- and intra-molecular interactions can be observed; only the cross-peaks of interest were circled in green. Dotted green circles identified cross-peaks slightly overlapped with the diagonal. Blue and red color represented negative- and positive-phase signals, respectively. Lowercase letters **a**–**f** were used for IDE proton assignment (see Figure 4).

With the aim to study the supramolecular interaction and binding sites between IDE and ACyD8, ^1^H NMR studies and 2D-ROESY experiments were performed (Figure 4 and Figure 5). NMR spectroscopy is one of the most useful tools for a structural analysis of the host/guest interaction, providing useful information on supramolecular systems. Figure 4 shows a comparison between the ^1^H NMR spectrum of the free drug (blue trace), ACyD8 (red trace) and the IDE/ACyD8 inclusion complex (green trace). The IDE/ACyD8 inclusion complex did not show separate signals for the free and complexed IDE as a consequence of the fast exchange between the free and included forms.

Although in the ^1^H NMR spectrum of the complex, the typical signals of IDE appeared to be unaffected by the interaction with ACyD8, some slight changes could be observed for all the ACyD8 proton resonances compared to the free macrocycle, pointing out their direct involvement in drug complexation.

To obtain further insights into the complexation, a ROESY study on the IDE/ACyD8 inclusion complex was performed with the aim to highlight the interaction sites of IDE with ACyD8 (Figure 5). In the ROESY spectrum of the IDE/ACyD8 complex, cross-peaks between the CH_3_ protons b of IDE and the signals of ACyD8 at 4.9 ppm (anomeric H-1) and at 2.3 ppm (CH_2_C(O) of octanoyl chains) were found. Furthermore, the cross-peak between the methoxy protons a,a′ of IDE (4 ppm) and the internal H-3/H-5 protons of ACyD8 indicates the close interaction of the IDE quinone ring with the ACyD8 cavity, while the IDE alkyl chain appeared to be aligned with the tethered octanoyl chains. ROE correlations were also found between the CH_2_ protons f of IDE (3.6 ppm) and the H-5 protons of the ACyD8 cavity, and between the CH_2_ protons e of IDE (1.5 ppm) and the H-6 protons of CyD surrounding the narrow rim.

Altogether, these findings evidenced the entanglement of IDE into ACyD8, pointing out selective supramolecular interactions of the drug with specific portions of ACyD8 (i.e., H-1, H-3, H-5, H-6, CH_2_C(O) of octanoyl chains at the wide rim).

#### 2.1.3. Molecular Modeling

**Figure 6:**
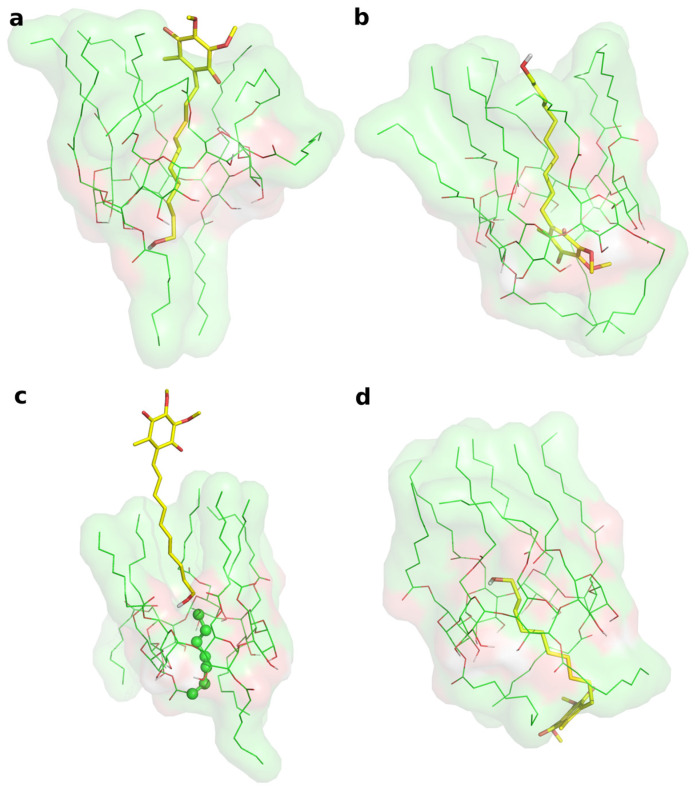
(**a**) Representative structure of the most populated cluster from the MD simulation of complex1. (**b**) Representative structure of the most populated cluster from the MD simulation of complex2. (**c**) MD snapshot of complex1 at 348 ns of simulation time. (**d**) Representative structure of the second ranked (by population) cluster from the MD simulation of complex2. In every panel, IDE is represented by yellow thick sticks, and ACyD8 is represented by green thin sticks and molecular surfaces.

Two different bound conformations of the inclusion complex were built in Maestro by manually docking IDE into the ACyD8 cavity, as in a previously reported approach [47]: in the first complex, the IDE ring leaned from the narrower rim (complex1—Figure 6a), whilst in the second one, it leaned from the wider rim (complex2—Figure 6b).

**Figure 7:**
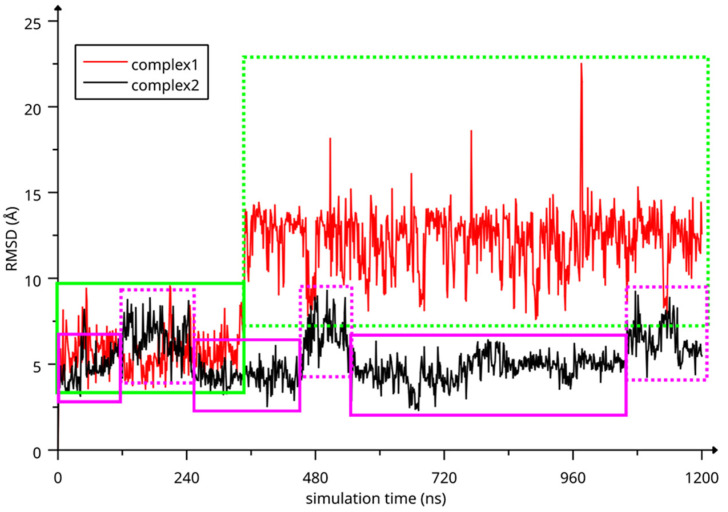
RMSD values of IDE atoms as a function of simulation time. The highlighted regions represent: IDE included in ACyD8 cavity (complex1—green solid line), IDE randomly interacting with the outer part of ACyD8 (complex1—green dashed line), cluster 1 of complex2 MD conformations (magenta solid lines) and cluster 2 of complex2 MD conformations (magenta dashed lines).

The two complexes were submitted to molecular dynamics (MD) simulations in an explicit solvent to challenge the complex conformations and to obtain insights into the host/guest interaction. Complex1 and complex2 showed different behaviors during the MD simulations. Figure 7 reports the atomic Root Mean Square Deviation (RMSD) values of IDE in complex1 (red line) and complex2 (black line) as a function of the simulation time. In the case of complex1, IDE remains in the ACyD8 cavity for 348 ns, and in the 0–348 ns simulation time range (Figure 7—green solid line), the inclusion complex conformations are found in the most populated cluster (286 conformations out of 1000—Figure 6a); then, IDE leaves the cavity and starts to randomly interact with the outer part of ACyD8 (Figure 7—green dashed line). Upon IDE unbinding, the ACyD8 cavity becomes occupied by an octanoyl lipophilic tail from the narrow rim (Figure 6c—green spheres); however, during the simulation, the tail becomes displaced by other octanoyl chains from the wide rim, giving rise to the self-inclusion phenomena that we previously reported for similar systems [42,43]. In the case of complex2, IDE remains in the ACyD8 cavity throughout the simulation time. The clustering of the inclusion complex conformations reveals that the trajectory can be clustered into the 2 most populated clusters, with the first one including 672 conformations out of 1000 (Figure 6b) and the second one including 264 conformations out of 1000 (Figure 6d). The magenta rectangles in Figure 7 indicate the simulation time ranges, where the two most populated clusters are found during the simulation (solid lines—cluster 1; dashed lines—cluster 2).

**Figure 8:**
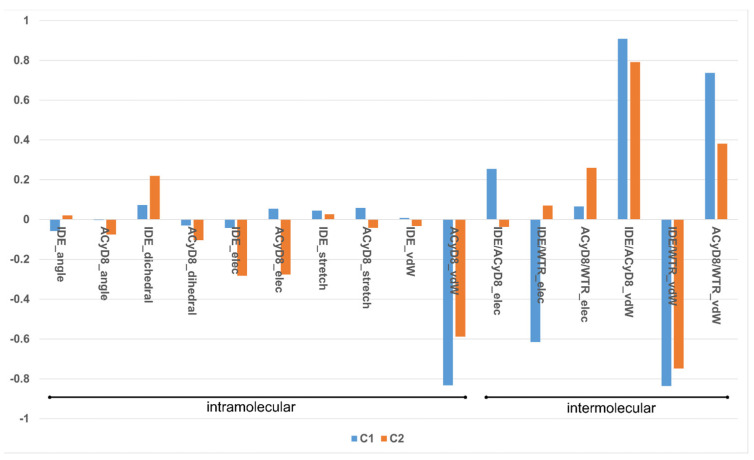
Plot of the correlation coefficients between IDE RMSD and force-field energy terms.

We studied the relationship between the motions of IDE during the MD simulations of complex1 and complex2 and the inter- and intra-molecular energetics by calculating the linear correlation coefficients between the RMSD values and the energy contributions. The force-field terms for the bonded energies of ACyD8 and IDE included angle, dihedral and stretch energies, whilst the non-bonded internal energies included van der Waals and electrostatic energies. The interaction energies included van der Waals and electrostatic intermolecular IDE/ACyD8, IDE/water and ACyD8/water energies (Figure 8).

**Figure 9:**
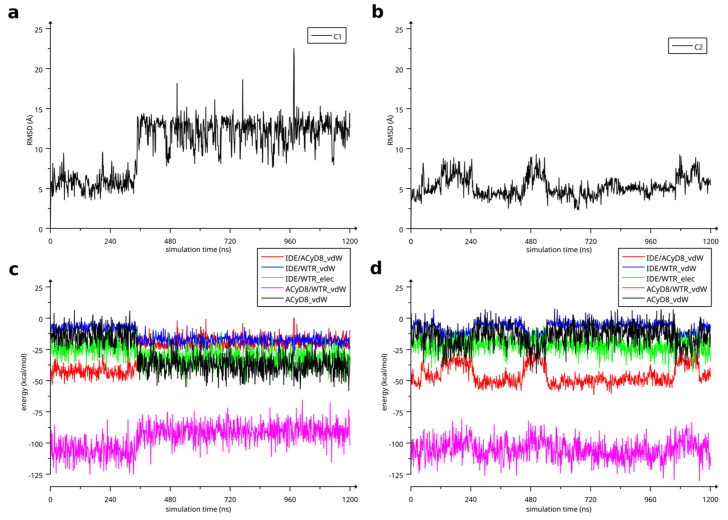
(**a**) RMSD of IDE atoms in complex1 as a function of MD simulation time; (**b**) RMSD of IDE atoms in complex2 as a function of MD simulation time. (**c**) Energetics of simulated complex1 as a function of simulation time. (**d**) Energetics of simulated complex2 as a function of simulation time.

Among the intramolecular energy contributions, the only term showing a high correlation with the RMSD values is the van der Waals energy of ACyD8. As reported in Figure 9, ACyD8_vdW becomes more favorable in both complex1 and complex2 when IDE moves away from ACyD8. In complex1, this is due to an improved interaction between the ACyD8 octanoyl tails and between these latters and the ACyD8 cavity, while in the case of complex2, the cavity always interacts with IDE; thus, ACyD8_vdW never becomes as negative as that in complex1. Among the intermolecular energy contributions, the highest RMSD correlation values are observed for the three van der Waals energy terms related to the IDE/ACyD8, IDE/WTR (water) and ACyD8/WTR interactions in both simulated inclusion complexes. In complex1, the loss of favorable IDE/ACyD8 and the ACyD8/WTR van der Waals interaction energy observed along with the unbinding of IDE is counterbalanced by the energy changes in the IDE/WTR_vdW, IDE/WTR_elec and ACyD_vdW terms. Although no IDE unbinding from ACyD8 is observed in complex2, the energy changes show similar patterns. It is worth noting that the IDE/ACyD8 intermolecular van der Waals term is much more favorable in complex2 than in complex1, suggesting an optimal IDE/ACyD8 interaction in complex 2, probably because of a better accommodation of the IDE ring next to the wide rim rather than next to the narrow one as in complex1.

### 2.2. Preparation and Characterization of IDE/ACyD8-NPs

#### 2.2.1. Nanoformulation and Physical—Chemical Characterization

IDE/ACyD8-NPs were prepared using the nanoprecipitation method. Firstly, we prepared blank NPs by using different ACyD8 concentrations to identify the optimum formulation in terms of size and yield. In Table 1, the yield percentage, the hydrodynamic radius (R_H_) and the polydispersity index (P.I.) of the realized formulations are reported. All the colloidal suspensions showed a progressive increase in R_H_ from 20 nm to 55 nm as the concentration of ACyD8 increased, without losing dimensional homogeneity (see P.I. in Table 1). The percentage yield (yield %) was also influenced by the amount of macrocycle used for the preparation of the NPs, increasing progressively with the increase in the ACyD8 concentrations (about 70% at the highest ACyD8 amount employed). The ACyD8-NPs showed negative zeta potential (ζ) values, suggesting good stability of the colloidal suspensions.

Based on its high yield % and R_H_, suitable for intranasal administration, the formulation prepared starting from 1 mM ACyD8 was selected for further studies. Thus, three different formulations (i.e., A, B and C in Table 2) were realized by using ACyD8 (1 mM) and three different amounts of IDE (0.5, 1 and 2 mM). The properties of the realized formulations are reported in Table 2.

A non-significant variation in the yield % and size was observed for all formulations, except for the sample prepared using the highest IDE theoretical concentration (sample C). In this case, sizes double those of the blank NPs prepared starting with the same ACyD8 amount were observed (1 mM), but this did not affect the therapeutical objective. A high E.E. % and D.L. % of IDE were observed for all formulations, confirming the high affinity of the IDE molecules for amphiphilic ACyD8, as already demonstrated by the IDE/ACyD8 interaction studies. All formulations showed more negative ζ values than the blank ACyD8-NPs (about −11 mV), and they were particularly high at the highest IDE theoretical concentration. This trend can demonstrate that IDE is located not only within the NP matrix but also on the surface, making the NP zeta potential more negative.

**Figure 10:**
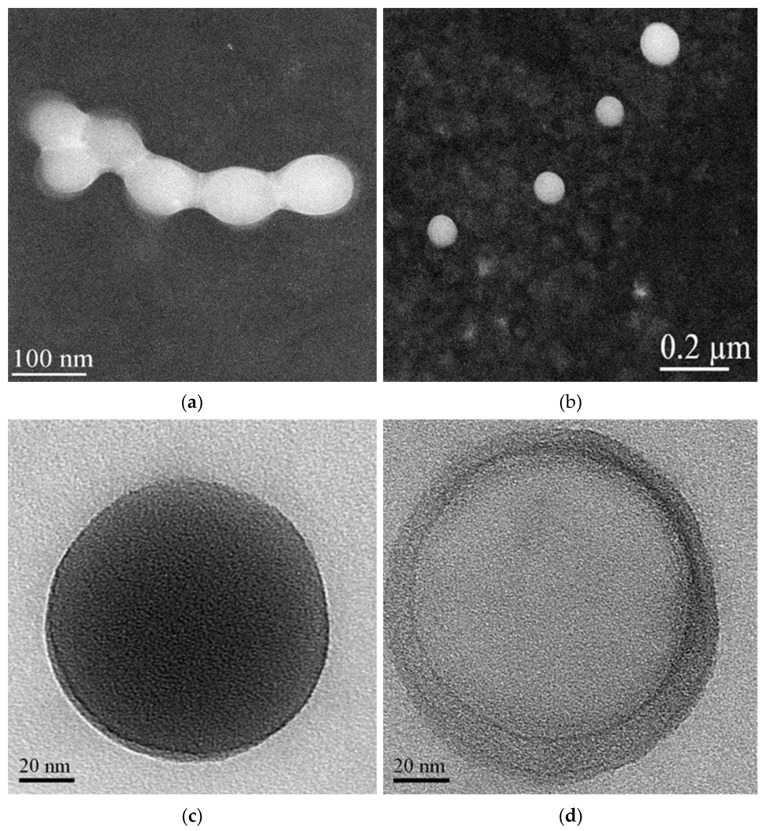
STEM images of lyophilized IDE/ACyD8-NPs before (**a**) and after redispersion (**b**). The NPs observed at higher magnification showed both matrix structure (**c**) and nanoreservoir structure (**d**).

A morphological characterization of the IDE/ACyD8-NPs was performed using scanning transmission electron microscopy (STEM) on the lyophilized powder and after its redispersion (Figure 10). The powder shows aggregates in which each single NP appears surrounded by a solid film (Figure 10a), probably constituted by the trehalose added before the lyophilization as a lyoprotectant agent. This allows for separate particles to be obtained after the redispersion of the IDE/ACyD8-NP powder. As observed in Figure 10b, the NPs are spherical, non-aggregate and present smooth surfaces.

After increasing the magnification, we observed particles that present different morphologies. Notably, we observed NPs with a dense structure (see Figure 10c for an example). However, some NPs with a non-uniform structure are evident, in which we can identify a core surrounded by a shell with different densities, a nanocapsule structure (Figure 10d). The literature data [25,48,49] indicate that non-ionic ACyDs can self-assemble in water, forming different structures, such as solid nanospheres or nanocapsules, based on different lengths of the acyl chain, the solvent used for nanoprecipitation or the presence of a surfactant. Furthermore, Choisnard et al. [50] demonstrated a homogeneously dense structure for nanoassemblies of hexanoate β-CyD, whilst multilayer vesicles were evidenced for decanoate β-CyD.

#### 2.2.2. FT-IR Analysis

**Figure 11:**
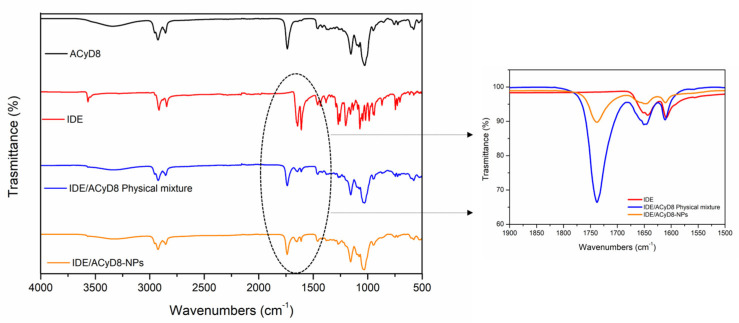
FT-IR spectra of IDE/ACyD8-NPs (sample C) compared to those of free components and the physical mixture. The inset shows magnification of the peaks in the range between 1750 cm^−1^ and 1600 cm^−1^, as highlighted by the dashed ellipse.

Figure 11 shows the FT-IR spectra of IDE, ACyD8, the IDE/ACyD8 physical mixture and the IDE/ACyD8-NPs prepared at a 2:1 molar ratio (sample C). For the pure drug, the main bands were observed at 3568 cm^−1^ (O-H stretching vibration); 2923 cm^−1^ and 2841 cm^−1^ (C-H stretching vibration); and 1652 cm^−1^, 1642 cm^−1^ and 1607 cm^−1^ (C=O and C=C stretching vibrations of the carbonyl ring). The ACyD8 spectrum showed the presence of a broad band from 3600 cm^−1^ to 3050 cm^−1^, reflecting the contribution to the O-H stretching vibration by different OH groups, attributable to primary (at 3525 cm^−1^) and secondary (at 3280 cm^−1^) OH groups. At lower wavenumbers, a double peak in the 2900–2859 cm^−1^ band (C-H stretching) and a prominent composite band at 1733 cm^−1^ (C=O stretching) were evident. The spectrum of the IDE/ACyD8 physical mixture is a superposition of the signals typical of IDE and ACyD8. However, a shift of the peak from 1733 cm^−1^, attributable to the C=O stretching of ACyD8, to 1737 cm^−1^ was observed (see the inset in Figure 11). Furthermore, the peak attributable to the C=O and C=C stretching vibrations of the IDE carbonyl ring slightly shifted to higher wavenumbers (from 1642 cm^−1^ and 1607 cm^−1^ to 1644 cm^−1^ and 1609 cm^−1^, respectively), probably as a consequence of the formation of hydrogen bonds between IDE and ACyD8 that could involve the external surface of the macrocycle. More intense shifts regarding the typical signals of the C=O and C=C stretching of IDE were observed in the spectrum of the IDE/ACyD8-NPs. They shifted from 1652 cm^−1^, 1642 cm^−1^ and 1607 cm^−1^ to 1656 cm^−1^, 1647 cm^−1^ and 1611 cm^−1^, respectively. These shifts, in combination with a lesser percentage of the intensity of the peaks in the IDE/ACyD8-NPs, confirm the successful inclusion of the drug into the macrocycle.

#### 2.2.3. Differential Scanning Calorimetry (DSC)

**Figure 12:**
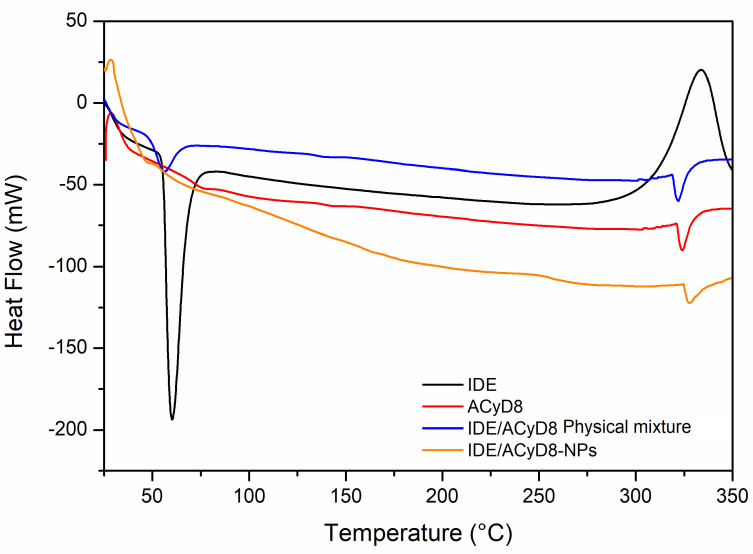
DSC traces of IDE/ACyD8-NPs (sample C), pure IDE and ACyD8, and the physical mixture.

The DSC curves of the IDE/ACyD8-NPs were obtained to determine the existence of drug/cyclodextrin interactions within the matrix network. Figure 12 shows the resulting DSC data for the IDE/ACyD8-NPs and their precursors. The DSC thermograms revealed an endothermic melting behavior with a peak T of 60 °C, which corresponds to the melting enthalpy of the pure drug, with degradation at a temperature around 330 °C. Three endothermic peaks are present in the ACyD8 sample at 75 °C, 143 °C and about 300 °C. The third peak, which is also present in the IDE/ACyD8-NP samples, can be attributed to the melting point of the systems. The physical mixture showed the superimposition of the two pure components. Furthermore, the disappearance of the exothermal peak due to the degradation of IDE is probably due to the protection exerted by ACyD8. A different thermogram was obtained for the IDE/ACyD8-NPs. In this trace, the endothermal signal of IDE is less evident than that of the physical mixture, and it is shifted to a slightly lower temperature, likely due to the inclusion/complexation of the drug in the NP network and/or due to the amorphous state of the systems. However, the exothermal peak observed at about 250 °C for the IDE/ACyD8-NPs evidences the sample’s transformation from an amorphous state to a crystalline state.

#### 2.2.4. In Vitro Release of IDE from the NPs

**Figure 13:**
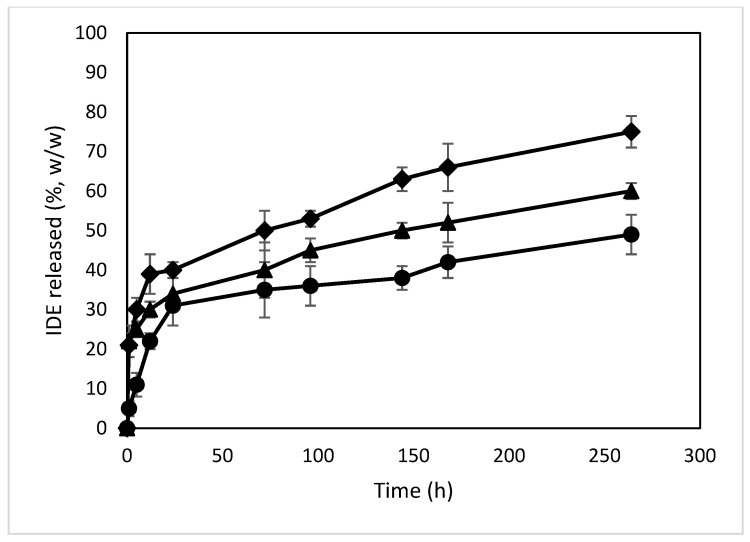
In vitro release profiles of IDE from IDE/ACyD8-NPs in PBS (pH 7.4). Sample A (circle), sample B (triangle), sample C (diamond).

The release profiles of IDE from the different formulations were obtained via dialysis in PBS (pH 7.4) and compared to those of the free drug (Figure 13). Free IDE crosses through the synthetic membrane within 60 min, confirming the suitability of the synthetic bag to carry out the experiment. All formulations showed a biphasic profile with a burst effect higher for the NPs with higher loading amounts of IDE (about 33%, 34% and 46% (*w/w*) within 24 h of the experiment for the formulations named A, B and C, respectively). This effect demonstrates the presence of IDE on the surface or in proximity to the surface of the NPs, thus characterized by a quick release and confirming the enhancement of the negative ζ value observed for the NPs when increasing the theoretical amount of IDE. After that, the NPs produced a sustained release of the drug that was extended for more than 11 days. This trend is due to the high affinity of IDE for ACyD8 and demonstrates the suitability of the ACyD8 NPs as a carrier for IDE delivery.

#### 2.2.5. Biological In Vitro Studies on SH-SY5Y Cells

**Figure 14:**
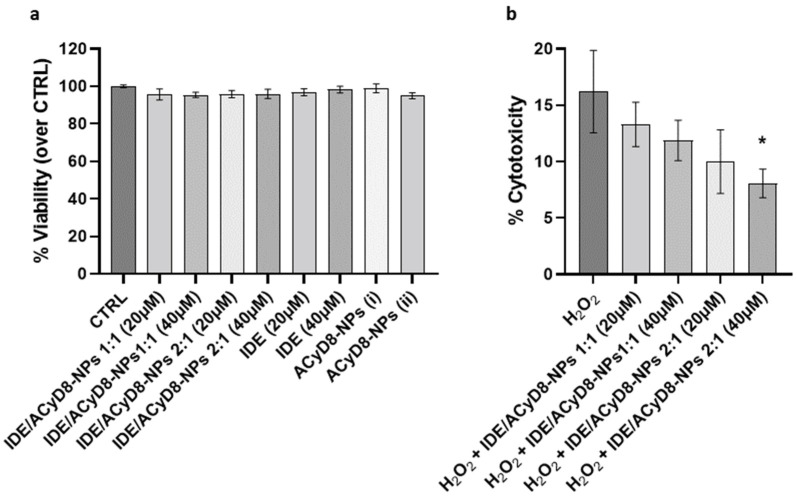
(**a**) Analysis of cell viability of neuronal SH-SY5Y cells treated with IDE/ACyD8-NPs, IDE and ACyD8 for 72 h. Data are presented as mean ± S.D. (**b**) Analysis of cytotoxicity on the same cells pretreated with IDE/ACyD8-NPs and exposed to 30 µM H_2_O_2_ for 72 h. Concentrations in IDE/ACyD8-NPs refers to the actual loading of IDE within NPs. Unloaded ACyD8-NPs (i) and (ii) were tested at the same ACyD8 concentration used for testing the drug-loaded NPs 20 µM and 40 µM, respectively. Data are presented as mean ± S.D. One-way ANOVA with Tukey’s multiple comparison test: in (**a**), all data are not statistically different; in (**b**), * *p* < 0.05 (H_2_O_2_ + IDE/ACyD8-NPs 2:1 40 µM versus H_2_O_2_).

After evaluating the release efficiency of IDE from the NPs, their effects on cultured neuronal SH-SY5Y cells were investigated. First, cell viability was analyzed in the presence of the IDE/ACyD8-NPs prepared at 1:1 and 2:1 molar ratios; moreover, the effects of IDE and the ACyD8-NPs alone were tested. For all compounds, two concentrations (i.e., IDE 20 µM and IDE 40 µM) were tested upon 72 h of incubation, where the IDE release was between 35 and 50% (*w/w*). The results in Figure 14a show that the NPs at both molar ratios and concentrations did not significantly affect the viability of the SH-SY5Y cells, confirming their suitability for in vivo approaches. The same results were obtained with IDE and the ACyD8-NPs alone.

To assess the potential antioxidant effects of the IDE/ACyD8-NPs in the presence of ROS, cytotoxicity was evaluated under 30 µM H_2_O_2_ treatment for 72 h (Figure 14b). The analysis of LDH release showed that H_2_O_2_ alone induced 16% cytotoxicity in the SH-SY5Y cells. Notably, the pretreatment with the IDE/ACyD8-NPs counteracted the H_2_O_2_ damage, with the 2:1 molar ratio at 40 µM significantly reducing 50% (*p* = 0.015) of the level of cytotoxicity. These data further support the ability of the ACyD8-NPs to efficiently release functional IDE over time.

## 3. Materials and Methods

Idebenone (IDE; 2,3-dimethoxy-5-methyl-6(10-hydroxydecyl)1,4-benzoquinone, C19H30O5, MW 338.44 Da) was a Sigma-Aldrich (Milan, Italy) product. ACyD8 was synthesized according to the literature [51]. All solvents were of analytical grade. Water was double-distilled and de-ionized, and then it was filtered through 0.22 μm Millipore^®^ GSWP filters (Bedford, MA, USA).

### 3.1. ACyD8/IDE Interaction Studies

#### 3.1.1. UV-Vis Titration Studies

UV-vis spectra were obtained in the 200–600 nm spectral range using a double-beam spectrophotometer (FullTech Instruments, mod PG T80—Roma, Italy). Free IDE (1 mM), or in the presence of increasing ACyD8 concentrations (from 0 to 2 mM), was solubilized in ethanol (1.185 mL) and added drop by drop to water (3.815 mL). The obtained suspensions were characterized by UV-vis.

UV-vis data were employed to determine the apparent binding constant (Kc) of the IDE/ACyD8 inclusion complex by means of the Benesi–Hildebrand equations given below [45,52].

1/(A-A_0_) = 1/(Δε Kc [ACyD8]^n^) + 1/Δε (1)

where A-A_0_ is the difference in the absorbance of IDE in the presence and absence of the macrocycle, Δε is the molar absorption coefficient of IDE, and Kc is the association constant. The value of (n) describes the stoichiometry of the complex. A linearity of the double-reciprocal plot by putting n = 1 in the equation suggests a 1:1 stoichiometry, whilst a linearity observed for n = 2 suggests the presence of a 1:2 (drug:CyD) inclusion complex. The ratio of the intercept on the slope of the straight line obtained by plotting 1/A-A_0_ vs. 1/[ACyD8]^n^ can be taken as an estimation of Kc [53].

#### 3.1.2. Molecular Modeling

Following previously reported information [54], CyD was modeled in Maestro [55] upon the crystal structure of β-cyclodextrin (CCDC entry 762697) [56] by the substitution of C6 oxygens of residues 1–7 and of C2 oxygens of residues 1, 3 and 7 with octanoyl groups. The IDE 3D structure was downloaded from Pubchem [57] (ID 3686).

Two inclusion complexes were built in Maestro by manually docking IDE into the CyD cavity: in the first one (complex1), the IDE ring leaned from the narrow rim, whilst in the second one (complex2), it leaned from the wide rim. Complex1 and complex2 were used to build two simulated environments, which were set up and run using Desmond [58]. The OPLS2005 [59] force field was used, treating salvation explicitly through the TIP3P water model [60]. Prior to the MD production stage, the systems were relaxed using a previously reported relaxation protocol [61], and then the systems were simulated for 1.2 μs at 300 K in the isothermal–isobaric ensemble, using a Nose–Hoover chain thermostat and a Martyna-Tobias-Klein barostat (1.01325 bar), with time steps set to 2 fs, 2 fs, and 6 fs for bonded, near and far interactions, respectively. The structures were sampled every 1.2 ns, and the trajectories were analyzed using VMD [62] and Maestro [55].

MD snapshots from the MD simulations were clustered according to their atomic RMSD values, using average linkage and a merge distance cutoff of 4 Å. IDE, and ACyD8 heavy atoms were used as reference atoms for fitting, whilst IDE heavy atoms were used for the RMSD matrix calculation. The same atom sets were used for the RMSD calculations reported in figures MM1 and MM4.

Correlations between the RMSD values and the force-field terms were calculated using the Pearson correlation coefficient as follows:

(2) $$\rho \left(X,Y\right)=\frac{cov(X,Y)}{\sigma_{X}\cdot \sigma_{Y}}=\frac{\sum_{i=1}^{n}\left({(x}_{i}-{µ}_{X}\right)\cdot \left(y_{i}-{µ}_{Y}\right)}{\sqrt{{\sum_{i=1}^{n}\left(x_{i}-{µ}_{X}\right)}^{2}}\cdot \sqrt{{\sum_{i=1}^{n}\left(y_{i}-{µ}_{Y}\right)}^{2}}}$$

(3) $${µ}_{X=}\frac{1}{n}\cdot \sum_{i=1}^{n}x_{i}\;{µ}_{Y=}\frac{1}{n}\cdot \sum_{i=1}^{n}y_{i}$$

Prior to correlation estimation, data points were interpolated as follows:

(4) $$X_{I}=\left\{x_{1},\frac{x_{1}+x_{2}}{2},\frac{x_{2}+x_{3}}{2}\ldots \frac{x_{n-1}+x_{n}}{2}\right\}Y_{I}=\left\{y_{1},\frac{y_{1}+y_{2}}{2},\frac{y_{2}+y_{3}}{2}\ldots \frac{y_{n-1}+y_{n}}{2}\right\}$$

#### 3.1.3. NMR Studies

^1^H NMR and 2D ROESY spectra were recorded on Varian 500 MHz and Agilent 500 MHz spectrometers at room temperature (r.t. ≊ 25 °C). The chemical shifts (δ) are expressed in ppm. ROESY spectra were recorded using the ROESYAD sequence (Rotating-Frame Overhauser Effect SpectroscopY with Adiabatic Pulse) with a mixing time of 200 ms. NMR spectra were recorded in CDCl_3_, taking into account that the IDE/ACyD8 complex is presumably preserved in CDCl_3_, according to the literature [63].

#### 3.1.4. Nanoparticle Preparation

Blank ACyD8 nanoparticles (ACyD8-NPs) were prepared by using the nanoprecipitation method [64]. Increasing concentrations of ACyD8, ranging from 0.1 mM to 1 mM, were solubilized in 1 mL of ethanol and added, at room temperature and under mechanical stirring, to 2 mL of water. The colloidal suspension that was immediately obtained was stirred at room temperature for 30 min; then, the organic solvent was removed under vacuum until a volume of 1.5 mL was obtained. The ACyD8-NP dispersions were added to 5% (*w/v*) trehalose as a lyoprotectant agent and freeze-dried for 72 h (VirtTis Benchtop K Instrument, SP Scientific, Gardiner, MT, USA).

To obtain IDE-loaded ACyD8-NPs (IDE/ACyD8-NPs), IDE was added at different concentrations (0.5, 1 and 2 mM) to an ethanolic solution containing 1 mM ACyD8. Thus, the same procedure used to prepare the blank ACyD8-NPs was used. After the remotion of the organic solvent, the colloidal suspensions were filtered through 0.22 μm Millipore^®^ PTFE filters (Bedford, MA, USA) to eliminate non-encapsulated IDE that precipitated because of the ethanol elimination. The filtered IDE/ACyD8-NPs suspensions were freeze-dried in the presence of 5% (*w/v*) trehalose. After that, the powders were maintained at room temperature and in the dark for the successive analysis.

#### 3.1.5. Characterization of the Nanoparticles

The blank ACyD8-NP and IDE/ACyD8-NP powders were collected and weighed to determine the yield % using the following formula:

Yield % = (Effective yield/Theoretical yield) × 100(5)

The mean hydrodynamic diameter (R_H_) of the redispersed lyophilized NPs was determined via photon correlation spectroscopy (PCS) by using a Zetasizer Nano ZS (Malvern Instrument, Malvern, UK), utilizing a noninvasive back-scattering (NIBS) technique. The measurements were performed at room temperature (25 ± 1 °C) at an angle of 90° with respect to the incident beam. The diameter and the polydispersity index (P.I.) were determined as the mean of thirty determinations on three different batches, and the results are expressed as mean value ± standard deviation (S.D.).

The zeta potential values (ζ) of the NPs were determined by using a Zetasizer Nano ZS (Malvern Instrument, Malvern, UK), fixing the power of a He−Ne laser at 5.0 mW and λ = 633 nm. The lyophilized samples were redispersed and injected into the electrophoretic cell of the instrument. The ζ value was determined on the basis of the electrophoretic mobility using the Smoluchowsky equation and a nominal value of the Smoluchowsky constant equal to 1.5. Each reported value is an average of 10 experiments on 3 different batches ± S.D.

A morphological characterization of the lyophilized samples was carried out using scanning transmission electron microscopy (STEM) with a Jeol JEM 2010F (JEOL Ltd., Tokyo, Japan) apparatus at a 200 kV accelerating voltage.

#### 3.1.6. Encapsulation Efficiency and IDE Loading

The encapsulation efficiency (E.E.) and the drug loading (D.L.) percentage of the IDE/ACyD8-NPs were determined by solubilizing the samples in 1 mL of ethanol. The obtained solutions were opportunely diluted, and IDE was quantified by employing UV-vis spectroscopy, in the range of 200–400 nm, using the same apparatus previously described. The E.E.% and D.L.% were calculated using the following equations:

E.E. (%) = (Amount of IDE in NPs in mg/Theoretical IDE in mg) × 100(6)

D.L. (%) = (Amount of IDE in NPs/Weight of recovered NPs) × 100(7)

#### 3.1.7. FT-IR Analysis

The infrared spectra were obtained using a Two FT-IR Spectrometer (PerkinElmer Inc., Waltham, MA, USA) with the ATR method in the range of 4000–500 cm^−1^. The IDE/ACyD8-NPs were analyzed comparatively to free IDE, ACyD8 and the physical mixture containing the same amount of IDE and ACyD8 present in the NPs.

#### 3.1.8. Differential Scanning Calorimetry (DSC) Analysis

DSC analyses were carried out by using a TAQ500 instrument (TA Instruments, New Castle, DE, USA) under nitrogen flow at a rate of 100 mL/min, from 25 °C to 500 °C, with a heating rate of 5 °C/min. The analyses were performed on the same samples used for the FT-IR analysis.

#### 3.1.9. In Vitro Release of IDE from the NPs

The release studies of IDE from the NPs were performed via dialysis (Spectra/Por dialysis bags, 3500 MWCO—Spectrum Laboratoris, Inc., Los Angeles, CA, USA) at 37.0 ± 0.5 °C. Weighted amounts of the IDE/ACyD8-NPs (7.5 mg) were suspended in 1 mL of a phosphate buffer solution (PBS, pH 7.4) and poured into the bag. The dialysis bags were placed into a beaker containing 100 mL of an ethanol/PBS (pH 7.4) (30/70, *v/v*) mixture to ensure sinking conditions, and they were subjected to magnetic stirring at 100 rpm at 37.0 ± 0.5 °C. At fixed time intervals (1, 5, 12, 24, 72, 96, 144, 168, 264 h), the dialysis medium was collected and replaced with 100 mL of a fresh ethanol/PBS (pH 7.4) (30/70, *v/v*) mixture. The collected volume was evaporated under vacuum at 40.0 ± 0.5 °C. The residue was solubilized in 2 mL of ethanol, and IDE was quantified by employing UV-vis spectroscopy using the apparatus previously described. The experiments were performed in triplicate, and the results are expressed as mean value ± S.D.

#### 3.1.10. SH-SY5Y Culture and Treatment

SH-SY5Y cells were purchased from ICLC (Interlab Cell Line Collection, accession number ICLC HTL95013; obtained from European Collection of Authenticated Cell Cultures (ECACC)) and cultured as described in [65,66]. Brefly, 1.6 × 10^4^ cells were seeded in 96-well plates, and treated with the following compounds: (1) IDE/ACyD8-NPs 1:1, IDE 20 µM; (2) IDE/ACyD8-NPs 1:1, IDE 40 µM; (3) IDE/ACyD8-NPs 2:1, IDE 20 µM; (4) IDE/ACyD8-NPs 2:1, IDE 40 µM; (5) IDE, 20 µM; (6) IDE, 40 µM; (7–8) unloaded ACyD8-NPs. Unloaded ACyD8-NPs were tested at the same ACyD8 concentration used for testing the drug-loaded NPs

Cell viability was evaluated with the Cell-Titer-Blue Cell Viability Assay (Promega, Madison, WI, USA, G8080) after 72 h. For the cytotoxicity analysis, the cells were pretreated with NPs (conditions 1–4) for 4 h and then exposed to 30 µM H_2_O_2_. Cytotoxicity was evaluated with the LDH-Cytotoxicity Assay Kit II (Abcam, Cambridge, UK, ab65393) after 72 h.

## 4. Conclusions

In this work, we developed NPs based on ACyD8 that efficaciously entrapped the lipophilic drug IDE. The NPs were prepared by using the nanoprecipitation method in the nanometric range (about 100 nm) with a smooth surface and showing a double morphology, that is, a matrix and a nanoreservoir structure, as demonstrated in STEM images. A DSC analysis showed an amorphous structure of the NPs, which crystallize before melting. Solution studies demonstrated a high affinity of IDE for the ACyD8 cavity, showing an association constant of about 19,000 M^−1^ for the complex at a 1:1 molar ratio. NMR and molecular modeling studies demonstrated the inclusion of IDE into the macrocycle, with the quinone ring near the wide rim of ACyD8.

Due to the high affinity of IDE for ACyD8, a very slow release of the drug from the NPs was observed, and, after a burst effect of about 40%, the release was prolonged for over 10 days.

These preliminary studies demonstrated the potentiality of the new ACyD8-NPs synthesized here as delivery systems for IDE. Moreover, when applied to SH-SY5Y target cells over 72 h, the IDE/ACyD8-NPs did not affect cell viability. Interestingly, these novel NPs significantly reduced H_2_O_2_-induced cytotoxicity, further supporting the suitability of these delivery systems for more complex in vitro/in vivo studies that are currently in progress to confirm these observations.

## Figures and Tables

**Table 1:** Yield %, sizes, polydispersity index (P.I.) and zeta potential (ζ) of blank ACyD8 NPs.

ACyD8 Concentration (mM)	Yield % ± S.D.	R_H_ (nm) ± S.D.	P.I. ± S.D.	ζ ± S.D. (mV)
0.2	10 ± 5	22.25 ± 1.02	0.103 ± 0.009	−11.2 ± 2.6
0.4	21 ± 1	45.32 ± 2.56	0.124 ± 0.008	−10.9 ± 3.1
0.6	48 ± 9	46.98 ± 1.08	0.101 ± 0.009	−11.4 ± 2.5
0.8	58 ± 8	50.78 ± 2.36	0.203 ± 0.007	−11.3 ± 2.3
1	71 ± 16	55.96 ± 3.99	0.177 ± 0013	−11.6 ± 1.1

**Table 2:** Yield %, hydrodynamic radius (R_H_), polydispersity index (P.I.), zeta potential (ζ), encapsulation efficiency (E.E.) and drug loading (D.L.) of IDE/ACyD8-NPs prepared using different theoretical amounts of IDE ^1^. The results are expressed as mean value ± standard deviation (S.D.).

Samples	Theoretical Amount of IDE (mM)	Yield % ± S.D.	R_H_ (nm) ± S.D.	P.I. ± S.D.	ζ ± S.D. (mV)	E.E. % ± S.D.	D.L. % ± S.D.
A	0.5	66 ± 9	40.12 ± 9.65	0.254 ± 0.010	−22.7 ± 4.5	69.21 ± 9.21	7.83 ± 1.02
B	1	68 ± 12	53.5 ± 10.21	0.156 ± 0.017	−24.4 ± 7.0	59.77 ± 8.35	10.73 ± 0.95
C	2	70 ± 10	96.4 ± 13.24	0.164 ± 0.011	−29.0 ± 5.4	64.20 ± 6.45	21.12 ± 1.89

^1^ ACyD8 concentration was fixed at 1 mM for all formulations.

## Data Availability

Data are contained within the article.
